# Clinical characteristics and risk factors for septic shock in neonates with early-onset sepsis: a matched case–control study

**DOI:** 10.3389/fmed.2026.1883371

**Published:** 2026-09-23

**Authors:** Juan Liu, Jinhui Hu, Lei Li, Jiajia Zhou, Yajuan Wang, Zhaojun Pan

**Affiliations:** 1 Neonatal Medical Center, Affiliated Hospital of Yang Zhou University Medical College, Huai'an Maternal and Child Health Care Center, Huai'an, China; 2 Department of Clinical Laboratory, Affiliated Hospital of Yang Zhou University Medical College, Huai'an Maternal and Child Health Care Center, Huai'an, China

**Keywords:** neonate, prealbumin, risk factors, sepsis, shock, systemic immune-inflammation index (SII)

## Abstract

**Background:**

Early-onset neonatal sepsis (EOS) progresses rapidly, and the development of septic shock is accompanied by a marked increase in case-fatality. This study aimed to systematically characterize the clinical features of, and identify factors associated with septic shock in neonates with EOS.

**Methods:**

Neonates with EOS complicated by septic shock who were admitted to Huai'an Maternal and Child Health Care Center constituted the case group. Controls were non-shock EOS neonates hospitalized during the same period and meeting the same inclusion criteria, selected at a 1:2 case-to-control ratio matched on gestational age and birth weight. Clinical and laboratory variables were collected and compared between groups. Candidate variables were screened by LASSO regression and subsequently entered into multivariable logistic regression. The predictive performance of the resulting variables was evaluated by receiver operating characteristic (ROC) curve analysis with bootstrap internal validation.

**Results:**

A total of 43 neonates with EOS complicated by septic shock and 86 matched non-shock controls were included. The two groups were comparable with respect to sex, gestational age, birth weight, mode of delivery, and major perinatal factors (all *P* > 0.05). LASSO regression retained four variables with non-zero coefficients. In subsequent multivariable analysis, higher prealbumin (OR = 0.945, 95% CI: 0.898–0.994, *P* = 0.028) and higher systemic immune-inflammation index (SII; OR = 0.958, 95% CI: 0.930–0.992, *P* = 0.025) were independently associated with lower odds of septic shock, whereas a higher procalcitonin-to-prealbumin (PCT/prealbumin) ratio was independently associated with higher odds of septic shock (OR = 3.061, 95% CI: 1.244–7.531, *P* = 0.015). ROC analysis showed that prealbumin, SII, and the PCT/prealbumin ratio each had acceptable predictive performance, and their combination yielded an AUC of 0.851 (95% CI: 0.764–0.939), with a sensitivity of 83.53% and a specificity of 85.41%; the bootstrap-corrected AUC 0.811 and LOOCV AUC 0.804.

**Conclusions:**

Higher prealbumin and SII levels were inversely associated with septic shock in neonates with EOS, whereas a higher PCT/prealbumin ratio was positively associated with septic shock. These findings may provide additional insight into the inflammatory and metabolic characteristics associated with greater disease severity in this population.

## 1 Introduction

Sepsis is a systemic inflammatory response syndrome triggered by infection, defined pathologically by a dysregulated host immune response that leads to impaired tissue perfusion, circulatory failure, and multi-organ dysfunction, and it remains a major global public health challenge (1). Reported mortality from neonatal sepsis ranges from 11% to 19%, and survivors frequently experience neurodevelopmental delay and other adverse short- and long-term outcomes (2, 3). On the basis of timing of onset, neonatal sepsis is conventionally classified as early-onset (EOS; ≤72 h after birth) or late-onset (LOS; >72 h after birth). Compared with LOS, EOS evolves more rapidly, and a subset of affected infants deteriorate within hours to septic shock and multi-organ dysfunction syndrome (4).

The neonatal immune system is developmentally immature across multiple components of both innate and adaptive immunity. Neutrophils, macrophages, and T cells exhibit attenuated capacities for inflammatory signaling and pathogen clearance, leaving newborns with reduced defense against infection and an increased risk of dissemination (5, 6). In addition, neonatal sepsis often lacks the classical clinical signs observed in adults; early manifestations are typically non-specific—including temperature instability, lethargy, feeding intolerance, poor responsiveness, and mild respiratory abnormalities, which further complicates timely recognition and diagnosis (7).

Septic shock represents one of the most severe manifestations of neonatal sepsis and is strongly associated with elevated mortality and poor prognosis (4). The pathophysiology and clinical consequences of septic shock have been extensively investigated in adults and older children, with inflammatory cascades, circulatory dysregulation, and multi-organ involvement implicated as central drivers (8). However, in neonates, clinical manifestations are often nonspecific, and the relationship between laboratory parameters and shock occurrence lacks consistent evidence. Moreover, existing studies exhibit significant heterogeneity in sample selection, parameter definitions, and reporting methods (9). Against this background, the present matched case–control study examined key clinical and laboratory variables in neonates with EOS complicated by septic shock, with the aim of clarifying associated factors and informing future studies of neonatal risk stratification.

## 2 Materials and methods

### 2.1 Study design and participants

This was a single-center, retrospective case–control study. Eligible participants were neonates admitted to Huai'an Maternal and Child Health Care Center between 1 January 2019 and 31 December 2025 who fulfilled the diagnostic criteria for early-onset neonatal sepsis. Infants who subsequently developed septic shock formed the case group. Controls were non-shock EOS neonates hospitalized during the same period and matched to cases at a 1:2 ratio using nearest-neighbor matching within ±1 week for gestational age and ±150 g for birth weight. For each matched control, the corresponding index time was defined as the same postnatal age at which the matched case developed septic shock. Clinical and laboratory variables were compared between groups to identify factors associated with the development of shock.

The study was approved by the Ethics Committee of Huai'an Maternal and Child Health Care Center (approval No. 2026007). Written informed consent was obtained from the legal guardians of all participants. The study was conducted in accordance with the Declaration of Helsinki and its subsequent amendments.

### 2.2 Inclusion and exclusion criteria

Neonates were included if they were diagnosed with EOS within 72 h after birth and had baseline clinical and laboratory data available before shock onset (cases) or at the corresponding index time (controls). All gestational ages and birth weights were eligible, and controls were EOS neonates who did not develop septic shock. Exclusion criteria were: (1) primary immunodeficiency, chromosomal abnormalities, inherited metabolic disease, or major congenital malformations affecting circulatory or immune function; (2) severe birth asphyxia; (3) major surgery, blood-product transfusion, or other conditions likely to confound circulatory or immune assessment before baseline sampling; (4) death or transfer before baseline sampling within the first 24 h of life; and (5) missing key clinical or laboratory data precluding outcome assessment.

### 2.3 Diagnostic criteria and definitions

EOS was defined as sepsis occurring within 72 h after birth (4, 10). Septic shock was defined as sepsis with cardiovascular dysfunction despite adequate fluid resuscitation. Cardiovascular dysfunction was characterized by gestational-age–appropriate hypotension or the need for vasoactive support (dopamine >5 *μ*g/kg/min or epinephrine/norepinephrine at any dose), together with evidence of impaired perfusion, including base deficit >5 mEq/L, arterial lactate >2 times the upper limit of normal, urine output <0.5 mL/kg/h, or capillary refill >5 s (10, 11). Severe birth asphyxia was defined as a 1-min Apgar score ≤3 or a 5-min Apgar score ≤5, together with an umbilical arterial blood pH <7.0 (10). Because inflammatory biomarkers, particularly PCT, may show physiological postnatal variation during the first 72 h of life, EOS diagnosis was based on the overall clinical presentation and laboratory findings rather than on any single inflammatory marker alone.

### 2.4 Clinical data collection

Data were extracted independently from the hospital electronic medical record system by two investigators and cross-checked; discrepancies were adjudicated by a third investigator. The variables collected included: (1) baseline and perinatal characteristics—sex, age at admission, age at symptom onset, birth weight, weight at admission, gestational age, mode of delivery, Apgar scores, maternal infectious history, and perinatal complications; (2) sepsis-related clinical manifestations—body temperature, lethargy, apnea, tachypnea, seizures, tachycardia, pathological jaundice, bulging fontanelle, and other infection-related signs; (3) major therapeutic interventions—fluid resuscitation, invasive mechanical ventilation (IMV) and its duration, parenteral nutrition, peripherally inserted central catheter (PICC) placement, antibiotic regimen, blood-product transfusion, and other supportive treatments; and (4) laboratory parameters—the first post-admission complete blood count, inflammatory markers, and biochemical indices, together with the corresponding derived composite indices. The laboratory variables retained in the final multivariable model were obtained before the onset of septic shock in cases and at the corresponding index time in controls.

Laboratory measurements were performed using standardized automated platforms in the hospital clinical laboratory. Routine biochemical parameters were measured using an ADVIA Chemistry XPT automated chemistry analyzer (Siemens Healthineers, Germany). Albumin was measured using ADVIA Chemistry Albumin BCP (ALBP) Reagent (Siemens Healthcare Diagnostics Inc., USA; REF 10711841) based on the bromocresol purple dye-binding method, and prealbumin was measured using ADVIA Chemistry PREALB Prealbumin Reagents (Siemens Healthcare Diagnostics Inc., USA; REF 03057680) by immunoturbidimetry. Procalcitonin (PCT) was measured using the Atellica IM BRAHMS Procalcitonin assay (Siemens Healthcare Diagnostics Inc., USA; REF 11202699) on an Atellica IM 1,600 automated chemiluminescence immunoassay analyzer (Siemens Healthineers, Germany), based on direct chemiluminescence. Cardiac troponin T was measured using the Cardiac Troponin T Assay Kit based on magnetic microparticle chemiluminescence (Xiamen Innodx Biotechnology Co., Ltd., Xiamen, China; product code TY21224652; Chinese registration No. 20142400052) on a Wan200 + automated chemiluminescence immunoassay analyzer from the same manufacturer. Complete blood counts and C-reactive protein (CRP) were measured using a BC−7500CS automated hematology analyzer (Shenzhen Mindray Bio-Medical Electronics Co., Ltd., Shenzhen, China), with CRP determined using the C-reactive Protein (CRP) Assay Kit based on latex-enhanced immunoscattering turbidimetry (Shenzhen Mindray Bio-Medical Electronics Co., Ltd., Shenzhen, China; Chinese registration No. 20202400893). All analyzers were routinely calibrated and maintained according to the manufacturers’ instructions. Internal quality control (IQC) was performed routinely in accordance with the laboratory's standard operating procedures, and the laboratory participated in external quality assessment (EQA) programs during the study period.

Calculation of derived indices. Using the first laboratory measurements obtained after admission, the derived indices were calculated as follows: NLR = neutrophil count/lymphocyte count; PLR = platelet count/lymphocyte count; MLR = monocyte count/lymphocyte count; SII = platelet count ×  neutrophil count/lymphocyte count; SIRI = neutrophil count ×  monocyte count/lymphocyte count; and PCT/prealbumin ratio = PCT/prealbumin. These indices were intended to provide an integrated reflection of inflammatory burden and host immune status in neonatal sepsis and have been used in previous neonatal studies (12, 13).

### 2.5 Statistical analysis

All statistical analyses were performed using SPSS, version 27.0. Continuous variables were first tested for normality; normally distributed data are presented as mean ± standard deviation and compared between groups using the independent-samples *t*-test, whereas non-normally distributed data are reported as median (interquartile range) and compared using the Mann–Whitney *U* test. Categorical variables are expressed as counts and percentages and compared using the *χ*² test or Fisher's exact test, as appropriate. Variables clearly measured after shock onset or directly related to the outcome definition were excluded from predictor selection. Missing data were handled using multiple imputation before subsequent statistical analyses. Given the limited number of septic shock events, candidate reduction preceded LASSO. Variables available before shock onset or the corresponding index time were entered into LASSO if clinically relevant and *P* < 0.05 in univariate analysis; this threshold served only as a screening criterion. Eleven variables met these criteria. Continuous predictors were standardized, and *λ* was selected by 10-fold cross-validation. The minimum-error criterion was used (*λ*min = 0.095). Variables with non-zero coefficients at *λ*min were then entered into multivariable logistic regression. With 43 septic shock events and four variables entered into the multivariable model, the events-per-variable (EPV) ratio was 10.8, with *post-hoc* power of approximately 69% under the observed effect sizes. Associations between the selected variables and septic shock were assessed using multivariable logistic regression. Considering that IMV may be closely related to disease severity, an additional sensitivity analysis was performed by refitting the multivariable model without IMV, with prealbumin, SII, and the PCT/prealbumin ratio entered simultaneously. Discriminative performance was evaluated by ROC curve analysis, with optimal cut-off values determined using the Youden index; sensitivity, specificity, PPV, and NPV were calculated at the corresponding thresholds. Bootstrap internal validation was performed to quantify model optimism, and pairwise DeLong tests were used for AUC comparisons. To further evaluate model stability, ridge-penalized logistic regression with standardized predictors and leave-one-out cross-validation (LOOCV) were performed. All tests were two-sided, and *P* < 0.05 was considered statistically significant.

## 3 Results

### 3.1 Baseline clinical characteristics

During the study period, 51 neonates with EOS complicated by septic shock were admitted. After excluding 2 cases with complex congenital cardiac malformations, 2 cases with immunodeficiency or inherited metabolic disorders, and 4 cases with missing baseline clinical data, 43 neonates fulfilling the inclusion criteria were retained. Using 1:2 matching on gestational age and birth weight, 86 non-shock EOS neonates hospitalized during the same period were selected as the control group. The flow of participant selection and matching is shown in Figure 1.

**Figure 1:**
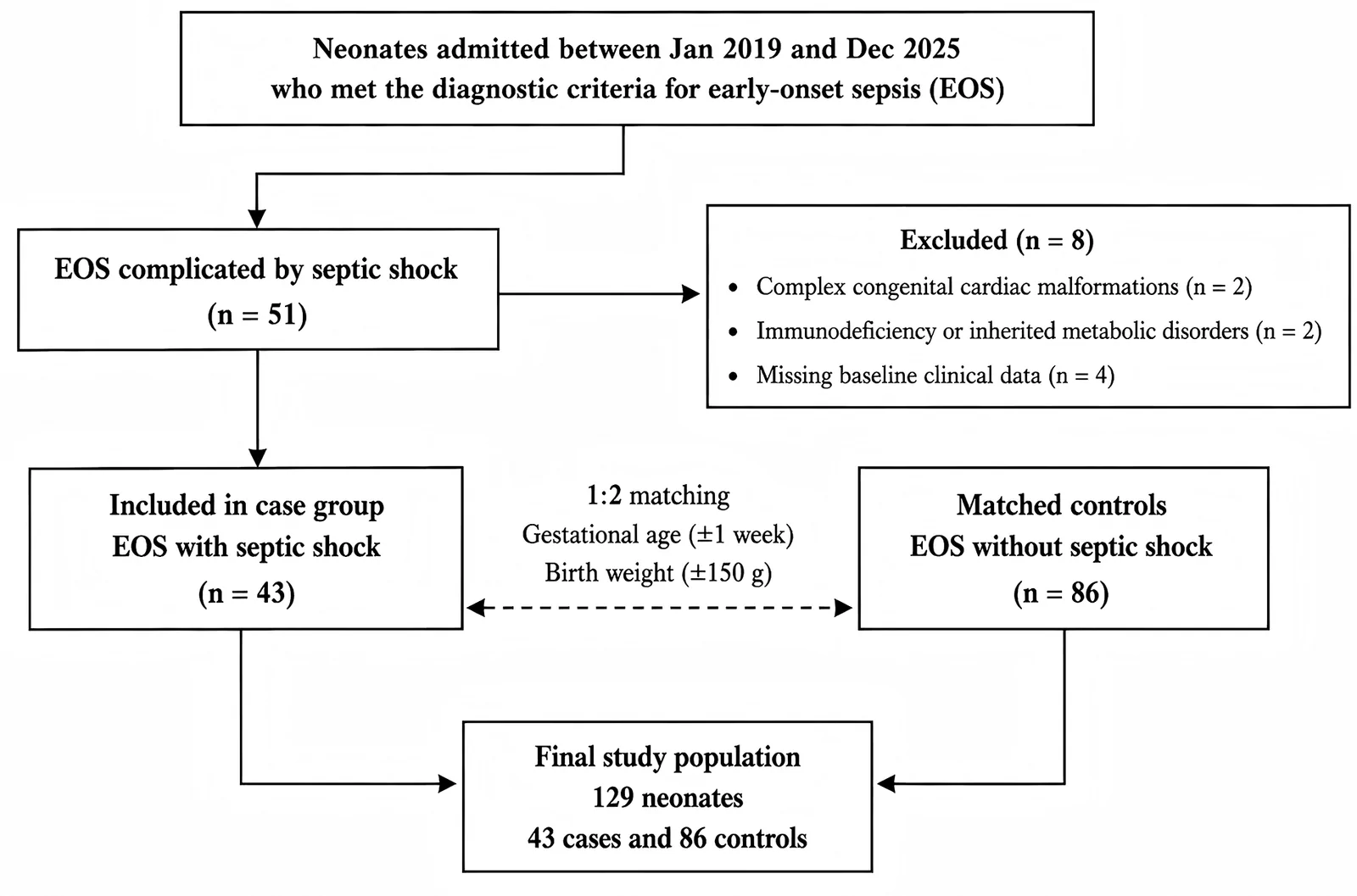
The study flowchart.

Baseline neonatal characteristics and maternal perinatal factors are summarized in Table 1. No statistically significant between-group differences were observed in sex, gestational age, birth weight, mode of delivery, perinatal asphyxia, prolonged rupture of membranes (≥18 h), small-for-gestational-age status, or patent ductus arteriosus (all *P* > 0.05). Maternal characteristics, including antepartum fever, chorioamnionitis, amniotic fluid contamination, gestational hypertension, and gestational diabetes likewise did not differ between groups (all *P* > 0.05).

**Table 1:** Clinical baseline characteristics of neonates with sepsis.

Variable	Non-shock group (*n* = 86)	Shock group (*n* = 43)	*P*
General neonatal information			
Male [%]	49 (57.0%)	26 (60.5%)	0.705
Female [%]	37 (43.0%)	17 (39.5%)	
Gestational age [weeks]	37 ^+^ ^3^ (32 ^+^ ^3^, 39 ^+^ ^4^)	37 ^+^ ^2^ (32 ^+^ ^1^, 39 ^+^ ^1^)	0.724
Birth weight [g]	2,975.00 (1,930.50, 3,528.50)	2,900.50 (1,875.00, 3,500.00)	0.836
Cesarean section [%]	33 (38.4%)	16 (37.2%)	0.898
Perinatal asphyxia [%]	6 (7.0%)	4 (9.3%)	0.907
Rupture of membranes ≥18 h [%]	34 (39.5%)	11 (25.6%)	0.117
Small for gestational Age [%]	9 (10.5%)	5 (11.6%)	1.000
Patent ductus arteriosus [%]	12 (14.0%)	7 (16.2%)	0.725
Maternal clinical characteristics during pregnancy			
Antepartum fever [%]	11 (12.8%)	3 (7.0%)	0.383
Chorioamnionitis [%]	6 (7.0%)	2 (4.7%)	0.606
Amniotic fluid contamination [%]	13 (15.1%)	11 (25.6%)	0.150
Gestational hypertension [%]	2 (2.3%)	1 (2.3%)	1.000
Gestational diabetes [%]	3 (3.5%)	0	0.550

Data are presented as median (IQR), or n (%), as appropriate.

### 3.2 Clinical manifestations and pre-shock interventions

Clinical manifestations and pre-shock major interventions are detailed in Table 2. With respect to clinical features, no significant differences were noted between groups in peak temperature, pallor, limb tremor, seizures, tachycardia, tachypnea, pathological jaundice, or bulging fontanelle (all *P* > 0.05). However, apnea (*P* = 0.007) and lethargy (*P* < 0.001) were significantly more frequent in the shock group than in the non-shock group. With regard to therapy, the shock group had a significantly higher rate of IMV (*P* = 0.023). Other supportive measures, including parenteral nutrition and PICC placement, did not differ significantly between groups (all *P* > 0.05).

**Table 2:** Clinical manifestations and main treatments of neonates with sepsis.

Variable	Non-shock group (*n* = 86)	Shock group (*n* = 43)	*P*
Clinical manifestations			
Fever peak [℃]	38.60 (38.20, 38.90)	38.05 (37.80, 38.55)	0.084
Pallor [%]	12 (14.0%)	10 (23.3%)	0.185
Shivering [%]	6 (7.0%)	4 (9.3%)	0.730
Seizures [%]	3 (3.5%)	4 (9.3%)	0.221
Tachycardia [%]	5 (5.8%)	2 (4.7%)	1.000
Tachypnea [%]	55 (64.0%)	28 (65.1%)	0.897
Apnea [%]	11 (12.8%)	14 (32.6%)	0.007
Pathological Jaundice [%]	37 (43.0%)	19 (44.2%)	0.900
Bulging fontanel [%]	2 (2.3%)	3 (7.0%)	0.332
Lethargy [%]	4 (4.7%)	12 (27.9%)	<0.001
Non-shock group			
IMV [%]	30 (34.9%)	24 (55.8%)	0.023
Parenteral nutrition [%]	44 (51.2%)	27 (62.8%)	0.211
PICC [%]	25 (29.1%)	16 (37.2%)	0.349

Data are presented as median (IQR), or n (%), as appropriate. IMV, invasive mechanical ventilation; PICC, peripherally inserted central catheter.

### 3.3 Laboratory findings at admission

Admission laboratory findings are compared between groups in Table 3. Among hematological parameters, platelet count (*P* < 0.001), PLR (*P* = 0.005), and SII (*P* = 0.010) were significantly lower in the shock group than in the non-shock group, whereas white blood cell count, absolute and relative neutrophil counts, absolute and relative lymphocyte counts, hemoglobin, red-cell distribution width, mean platelet volume, platelet distribution width, monocyte count, NLR, MLR, and SIRI did not differ significantly between the groups (all *P* > 0.05). Among biochemical indices, albumin (*P* = 0.006) and prealbumin (*P* = 0.004) were significantly lower, while troponin T (*P* = 0.026) and the PCT/prealbumin ratio (*P* < 0.001) were significantly higher, in the shock group than in the non-shock group. Other biochemical markers, including globulin, AST, ALT, alkaline phosphatase, cholinesterase, *α*-hydroxybutyrate dehydrogenase, lactate dehydrogenase, creatine kinase, creatine kinase isoenzymes, glycocholic acid, CRP, PCT, and the ratios of CRP and PCT to total protein, albumin, and prealbumin did not differ significantly between groups (all *P* > 0.05).

**Table 3:** Comparison of laboratory indicators in neonates with sepsis.

Variable	Non-shock group(*n* = 86)	Shock group(*n* = 43)	*P*
Hematological and Derived Indicators			
WBC [×10⁹/L]	12.49 (8.57, 20.33)	11.09 (6.78, 16.32)	0.231
Neutrophil count [×10⁹/L]	8.49 (5.00, 15.76)	6.260 (2.99, 13.50)	0.225
Neutrophil percentage [%]	64.81 ± 17.21	59.71 ± 22.12	0.154
Lymphocyte count [×10⁹/L]	2.70 (1.86, 3.77)	2.98 (1.69, 4.30)	0.772
Lymphocyte percentage [%]	20.50 (13.90, 31.10)	19.00 (11.55, 47.80)	0.836
Hemoglobin [g/L]	160.88 ± 32.43	155.72 ± 17.98	0.334
Red blood cell distribution width [%]	15.53 ± 1.26	15.89 ± 1.57	0.161
Platelet count [×10⁹/L]	241.00 (186.00, 291.00)	162.00 (86.00, 238.00)	<0.001
Mean platelet volume [fL]	9.80 (9.40, 10.80)	10.20 (9.80, 10.70)	0.135
Platelet distribution width [%]	14.30 (11.30, 16.50)	13.30 (11.10, 16.60)	0.925
Monocyte count [×10⁹/L]	1.13 (0.54, 1.76)	0.89 (0.48, 2.33)	0.813
NLR	3.51 (1.68, 5.78)	3.27 (0.84, 6.09)	0.418
PLR	79.22 (59.24, 126.79)	65.75 (31.25, 91.78)	0.005
MLR	2.75 (1.67, 4.28)	2.34 (1.46, 5.21)	0.572
SII	771.41 (363.24, 1,263.16)	463.06 (144.79, 924.45)	0.010
SIRI	3.36 (1.15, 8.44)	2.98 (0.55, 9.02)	0.818
Biochemical and derived indicators			
Albumin [g/L]	34.00 ± 4.88	31.55 ± 4.95	0.006
Globulin [g/L]	16.52 ± 3.81	15.50 ± 5.25	0.216
Prealbumin [mg/L]	52.30 (47.60, 61.00)	47.60 (42.00, 55.35)	0.004
AST [U/L]	57.30 (45.10, 91.15)	66.20 (51.50, 180.70)	0.063
ALT [U/L]	9.40 (5.90, 13.60)	14.80 (5.70, 29.40)	0.088
Alkaline phosphatase [U/L]	178.00 (128.50, 227.00)	173.00 (142.50, 220.00)	0.957
Cholinesterase [U/L]	5,058.00 (4,238.00, 5,892.00)	4,562.00 (3,966.00, 5,544.00)	0.091
*α*-Hydroxybutyrate dehydrogenase [U/L]	545.00 (448.00, 753.50)	588.00 (459.50, 897.00)	0.244
Lactate dehydrogenase [U/L]	688.90 (580.00, 1,030.00)	773.00 (606.50, 1,337.00)	0.180
Creatine kinase [U/L]	311.60 (190.40, 509.40)	308.90 (200.40, 777.00)	0.843
Creatine KINASE ISOENZYMES [U/L]	9.00 (6.00, 18.00)	9.25 (5.20, 12.70)	0.377
Troponin T [ng/L]	43.69 (11.12, 95.24)	97.87 (28.36, 267.60)	0.026
Glycocholic acid [mg/L]	4.15 (2.07, 9.23)	4.89 (1.94, 17.37)	0.544
CRP [mg/L]	4.88 (0.50, 21.48)	3.96 (0.50, 30.16)	0.833
PCT [ng/ml]	2.79 (0.58, 16.79)	14.12 (0.78, 47.04)	0.139
CRP/Total Protein	0.08 (0.01, 0.36)	0.09 (0.01, 0.58)	0.648
CRP/Albumin	2.33 (0.16, 28.29)	1.16 (0.33, 33.49)	0.740
CRP/Prealbumin	0.11 (0.01, 0.43)	0.14 (0.01, 0.89)	0.543
PCT/Total Protein	0.02 (0.01, 0.13)	0.07 (0.01, 0.37)	0.071
PCT/Albumin	2.03 (0.46, 9.50)	2.05 (0.51, 47.25)	0.406
PCT/Prealbumin	0.04 (0.01, 0.19)	0.56 (0.03, 1.14)	<0.001

Data are presented as mean ± SD or median (IQR), as appropriate. WBC, white blood cell count; NLR, neutrophil-to-lymphocyte ratio; PLR, platelet-to-lymphocyte ratio; MLR, monocyte-to-lymphocyte ratio; SII, systemic immune-inflammation index; SIRI, systemic inflammatory response index; CRP, c-reactive protein; PCT, procalcitonin.

### 3.4 Variable selection and multivariable regression analysis

Variables showing significant between-group differences in univariate analysis and judged to be clinically relevant were entered into LASSO regression (Figure 2). LASSO retained four variables with non-zero coefficients: prealbumin (−0.013), SII (−0.011), IMV (0.956), and the PCT/prealbumin ratio (0.018). These four variables were subsequently entered into the multivariable logistic regression analysis. In the multivariable model (Table 4), higher prealbumin (OR = 0.945, 95% CI: 0.898–0.994, *P* = 0.028) and SII (OR = 0.958, 95% CI: 0.930–0.992, *P* = 0.025) were independently associated with lower odds of septic shock, whereas a higher PCT/prealbumin ratio (OR = 3.061, 95% CI: 1.244–7.531, *P* = 0.015) was independently associated with higher odds of septic shock. The resulting combined model was expressed as logit (P) = 2.301−0.057 × prealbumin−0.043 × (SII/100) + 1.119 × PCT/prealbumin. Given the potential relationship between IMV and disease severity, a sensitivity analysis was performed after excluding IMV. Higher prealbumin (OR = 0.946, 95% CI: 0.901–0.993, *P* = 0.025) and higher SII (OR = 0.947, 95% CI: 0.899–0.998, *P* = 0.042) remained independently associated with lower odds of septic shock, whereas a higher PCT/prealbumin ratio remained independently associated with higher odds of septic shock (OR = 3.036, 95% CI: 1.241–7.426, *P* = 0.015). As a further assessment of coefficient stability, ridge-penalized logistic regression yielded standardized coefficients of −1.046 for prealbumin, −0.361 for SII, and 0.418 for the PCT/prealbumin ratio, respectively, preserving the direction of associations observed in the conventional multivariable model.

**Figure 2:**
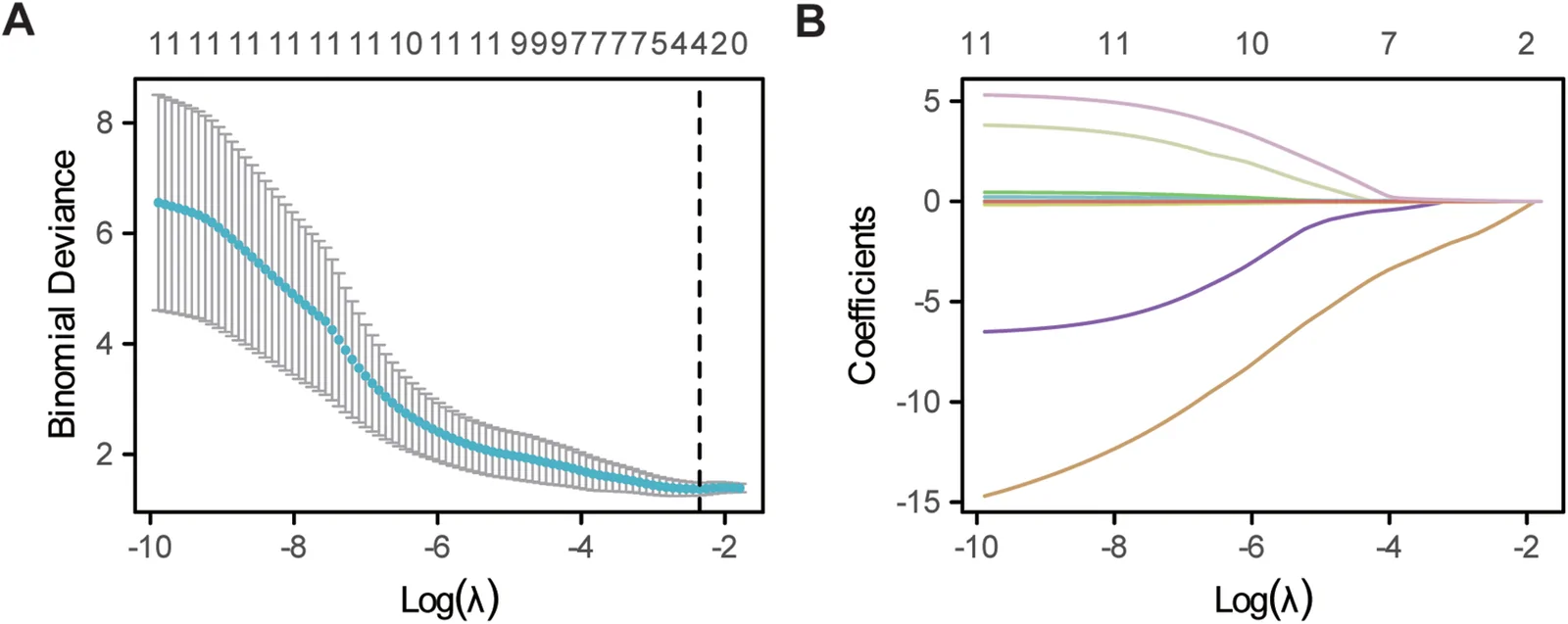
LASSO regression for selection of candidate predictors of septic shock in neonates with EOS. **(A)** Ten-fold cross-validation curve for selection of the optimal *λ*, with binomial deviance plotted against log(*λ*). **(B)** LASSO coefficient profiles of the candidate predictors as a function of log(*λ*).

**Table 4:** Univariate and multivariable logistic regression analyses of factors associated with septic shock.

Variable	Univariate OR (95% CI)	*P* value	Adjusted OR (95% CI)	*P* value
IMV	2.358 (1.117–4.975)	0.025	1.167 (0.426–3.193)	0.764
SII	0.998 (0.997–0.999)	<0.001	0.958 (0.930–0.992)	0.025
Prealbumin	0.953 (0.921–0.987)	0.007	0.945 (0.898–0.994)	0.028
PCT/prealbumin ratio	4.149 (1.842–9.346)	<0.001	3.061 (1.244–7.531)	0.015

OR, odds ratio; CI, confidence interval; IMV, invasive mechanical ventilation; SII, systemic immune-inflammation index; PCT, procalcitonin.

### 3.5 ROC curve analysis

The predictive performance of the selected variables for septic shock in EOS was assessed by ROC analysis (Table 5; Figure 3). Prealbumin yielded an AUC of 0.661 (95% CI: 0.559–0.762), SII an AUC of 0.741 (95% CI: 0.652–0.829), and the PCT/prealbumin ratio an AUC of 0.680 (95% CI: 0.562–0.798). The combined model yielded the highest AUC among the evaluated models, with an AUC of 0.851 (95% CI: 0.764–0.939). At the Youden-derived cut-off, the combined model showed a sensitivity of 83.53%, a specificity of 85.41%, a PPV of 74.11%, and an NPV of 91.21%. Bootstrap internal validation yielded an optimism-corrected AUC of 0.811. The AUC based on LOOCV predictions was 0.804. Pairwise DeLong testing showed that the combined model had a significantly higher AUC than the PCT/prealbumin ratio alone (AUC difference = 0.171, 95% CI: 0.008–0.234; *P* = 0.036), whereas the differences compared with prealbumin (*P* = 0.115) and SII (*P* = 0.093) were not statistically significant.

**Table 5:** ROC curve parameters for individual markers and the combined model.

Variable	Cut-off	AUC	95% CI	Sensitivity (%)	Specificity (%)	PPV (%)	NPV (%)
Prealbumin (per 1 mg/L)	47.20	0.661	0.559–0.762	48.84	68.25	43.48	72.74
SII (per 100 units)	653.45	0.741	0.652–0.829	60.00	79.07	58.90	79.81
PCT/prealbumin (per unit)	0.45	0.680	0.562–0.798	85.29	52.94	47.54	87.80
Combined Prediction	0.66	0.851	0.764–0.939	83.53	85.41	74.11	91.21

AUC, area under the curve; CI, confidence interval; PCT, procalcitonin; SII, Systemic immune-inflammation index.

**Figure 3:**
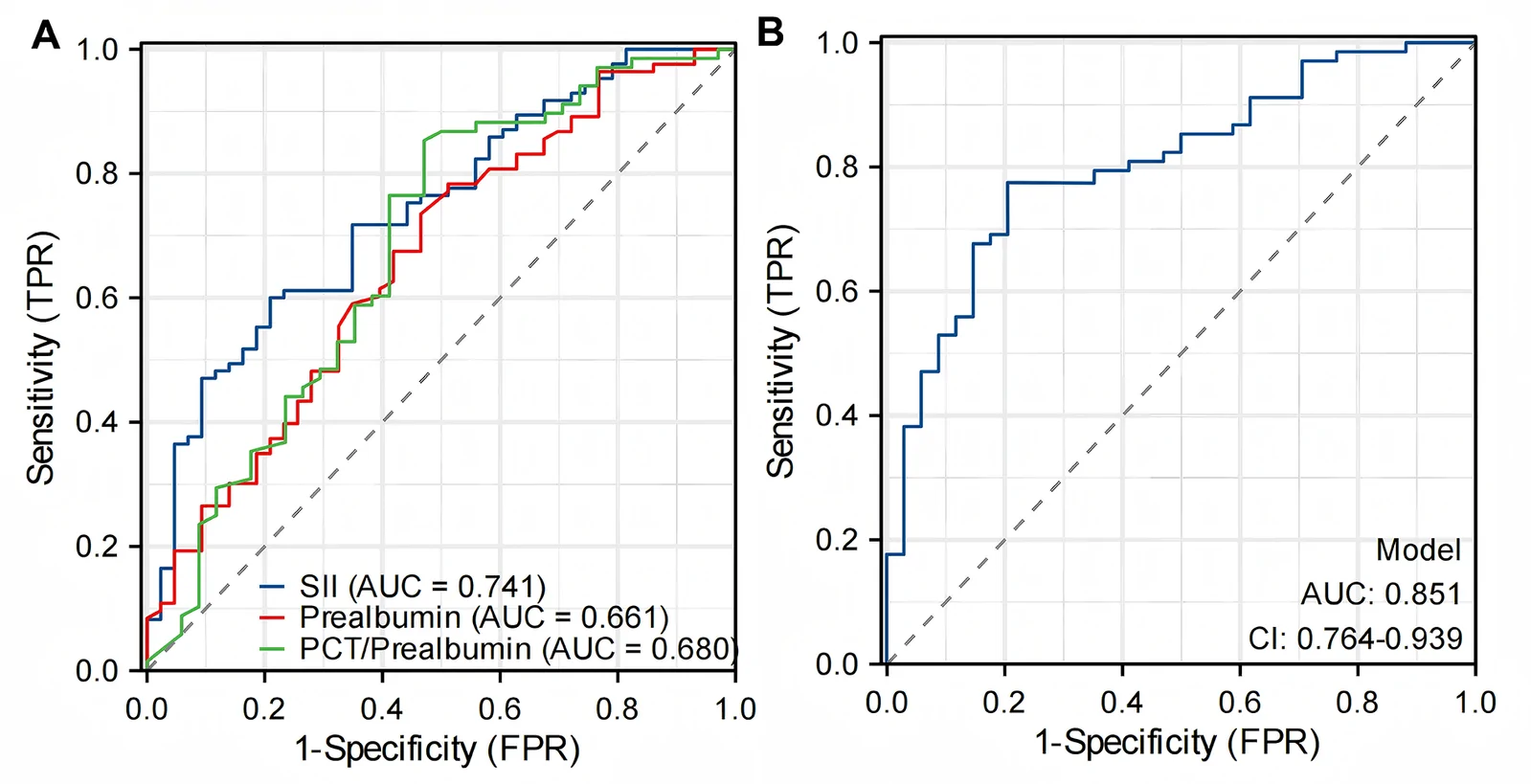
Receiver operating characteristic (ROC) curve analysis for prediction of septic shock in neonates with EOS. **(A)** ROC curves for prealbumin, SII, and the PCT/prealbumin ratio. **(B)** ROC curve for the combined model incorporating prealbumin, SII, and the PCT/prealbumin ratio.

## 4 Discussion

After matching for gestational age and birth weight, we used a combined LASSO and multivariable logistic regression strategy to systematically screen pre-shock clinical and laboratory variables and to identify indicators associated with septic shock in EOS. Higher prealbumin and SII levels were inversely associated with septic shock, whereas a higher PCT/prealbumin ratio was positively associated with septic shock. After full variable selection, prealbumin, SII, and the PCT/prealbumin ratio remained independently associated with septic shock in EOS.

Prealbumin is a low-molecular-weight protein synthesized by the liver. Its plasma concentration reflects not only nutritional supply but also modulation by inflammatory responses and acute-phase reactions (14). Because of its short half-life, plasma prealbumin responds to acute physiological perturbations more rapidly than albumin and is therefore considered a useful indicator of short-term shifts in protein metabolism (15, 16). In the present study, higher prealbumin levels were inversely associated with septic shock, which may reflect differences in protein-nutritional status, hepatic synthetic function, and the systemic response to infection (17, 18). These findings therefore support prospective evaluation rather than routine clinical use of a study-derived cut-off.

SII has attracted increasing attention as a composite marker reflecting both inflammatory and immune status. In critically ill adults with infection, elevated SII has generally been associated with greater inflammatory burden, more severe disease, and worse outcomes (19, 20). Evidence in neonates remains limited and is not directly comparable, as previous studies have mainly evaluated peripheral leukocyte ratios as biomarkers for neonatal sepsis or hematological inflammatory indices for pathogen differentiation in culture-proven late-onset neonatal sepsis (12, 13). In our EOS cohort, NLR did not differ between groups and PLR was lower in the shock group only in univariate analysis, whereas SII remained independently inversely associated with septic shock. Differences in study population, timing of sampling, sepsis onset, and outcome definition may partly account for these divergent findings. This discrepancy may be related to the distinct physiological and immunological characteristics of neonates, whose immune systems are developmentally immature, with predominant innate immunity and incompletely developed adaptive responses (21, 22). Under severe septic stress, neonatal hematopoiesis may be suppressed, leading to reductions in platelet and lymphocyte counts. At the same time, experimental and translational studies suggest that neonatal sepsis may evolve from an early inflammatory phase toward immune-cell dysfunction and immunosuppression (21, 23, 24). These changes could result in a decline in SII despite ongoing disease progression. In addition, hemodynamic deterioration may occur before an adult-like high-SII inflammatory phenotype becomes established. Together, these mechanisms may help explain the inverse association observed in our cohort, although they remain speculative and require confirmation in prospective studies with serial hematologic and immune measurements.

The PCT/prealbumin ratio integrates a conventional infection biomarker with an indicator of nutritional status, capturing both the intensity of infection-driven systemic inflammation and, indirectly, the balance between physiological stress and metabolic reserve (25, 26). Elevated PCT is associated with systemic bacterial infection, whereas reduced prealbumin may reflect altered protein metabolism and hepatic synthesis during acute illness. Previous studies in adults with sepsis have suggested an association between the PCT/prealbumin ratio and adverse outcomes (27). In our study, a higher PCT/prealbumin ratio was independently associated with septic shock in neonates with EOS, suggesting that the concurrent presence of a pronounced inflammatory response and impaired metabolic status may characterize more severe disease (28).

Taken together, the inflammatory cascade, regulation of immune-cell function, and nutritional and metabolic status interact in complex ways during neonatal sepsis, consistent with emerging evidence from other neonatal diseases highlighting the close interplay between metabolic homeostasis and immune regulation (29). Because the neonatal immune system is immature, with innate immunity dominant, adaptive immunity incomplete, and the release and regulation of inflammatory mediators not fully developed, inflammatory responses are prone to dysregulation. This dysregulation may compromise endothelial function, impair microcirculation, and reduce organ perfusion, ultimately facilitating progression to septic shock (21, 30). In light of these mechanisms, a shift in neonatal sepsis research from isolated laboratory markers toward composite indices that capture both systemic inflammation and nutritional burden may better align with the underlying pathophysiology. These neonatal-specific physiological characteristics also have implications for clinical management. Although the updated Surviving Sepsis Campaign pediatric guidelines provide guidance for the management of sepsis and septic shock, their application to neonates, particularly preterm infants, requires consideration of the distinct physiological characteristics of this population (9).

A strength of this study is the matching of cases and controls on gestational age and birth weight, which reduced potential confounding by these important perinatal characteristics. Nevertheless, several limitations should be acknowledged. First, the retrospective design and modest number of septic shock events limited statistical power and the stability of multivariable estimates. Moreover, univariate prescreening before LASSO may have excluded variables with weak marginal but potentially relevant joint effects, while predictor selection and model development within the same cohort may have introduced overfitting and optimistic post-selection estimates despite bootstrap internal validation. Given the potential relationship between IMV and disease severity, we additionally refitted the multivariable model after excluding IMV; the associations of prealbumin, SII, and the PCT/prealbumin ratio remained consistent with the primary analysis, providing some support for the robustness of the main findings. Second, reliance on electronic medical records may have introduced information bias because some clinical data were incompletely documented. Third, this was a single-center study without external validation, which may limit the generalizability of the findings. Therefore, these results should be considered exploratory and hypothesis-generating and require confirmation in larger prospective multicenter cohorts.

## 5 Conclusions

This study provides a systematic analysis of factors associated with septic shock in early-onset neonatal sepsis, identifying prealbumin, SII, and the PCT/prealbumin ratio as independently associated variables. The combined model showed moderate discrimination after internal bootstrap correction; however, the small single-center cohort, data-driven model development, and absence of external validation mean that these findings are hypothesis-generating. Prospective multicenter validation with serial biomarker sampling and prespecified cut-offs is required before these markers can be recommended for routine early risk stratification or clinical decision-making.

## Data Availability

The original contributions presented in the study are included in the article/Supplementary Material, further inquiries can be directed to the corresponding authors.
